# Bacteremia after Endoscopic Submucosal Excavation for Treating the Gastric Muscular Layer Tumors

**DOI:** 10.1155/2015/306938

**Published:** 2015-04-28

**Authors:** Guohua Li, Sheng Zeng, Youxiang Chen, Xiaojiang Zhou, Nonghua Lv

**Affiliations:** Department of Gastroenterology, The First Affiliated Hospital of Nanchang University, Nanchang, Jiangxi 330006, China

## Abstract

*Background*. The bacteremia is reported as being infrequent and transient in gastric EMR and ESD for treating gastric mucosa lesions or superficial gastric neoplastic lesion. There was no report of it being investigated in ESD for treating gastric muscular layer tumors (endoscopic submucosal excavation, ESE). This study aimed to determine the frequency of bacteremia in gastric ESE. *Patients and Methods*. A prospective study, in 122 consecutive patients who underwent gastric ESE for treating gastric muscular layer tumors, investigated the frequency of bacteremia before and 15 minutes after the procedure. *Results*. The median time for the total ESE procedure was 29 min (range from 8 to 62 min). The mean size of the biggest diameter of each resected specimen was 10 ± 2.7 mm (range from 5 mm to 30 mm). Blood cultures obtained before ESE were positive in 0% (0/122) of cases. Blood cultures obtained 15 min after ESE were positive in 2.5% (3/122) of cases. Six blood samples contained *Staphylococcus* with coagulase negative, which was considered contaminant. No signs of sepsis were seen in all patients. *Conclusions*. The frequency of bacteremia after gastric ESE was low. ESE for treating gastric lesions is thought to have a low risk of infectious complications; therefore, prophylactic administration of antibiotics may not be warranted.

## 1. Introduction

Bacteremia may develop endocarditis or other infection complications. As a result, the American Society for Gastrointestinal Endoscopy (ASGE), the American Heart Association (AHA), and other societies recommended prophylactic administration of antibiotics for high-risk patients undergoing high-risk procedures [1–6]. It has been known that there was an increased rate of bacteremia or local infection after some endoscopy procedures (such as esophageal sclerotherapy, esophageal stricture dilation, endoscopic retrograde cholangiopancreatography with biliary obstruction, endoscopic drainage of a pancreatic pseudocyst, endoscopic placement of feeding tubes, and EUS-FNA) [7–20]. So those guidelines recommended some endoscopy procedures should use prophylactically antibiotics in some high-risk patients. Until now, there were no formal recommendations regarding the need for prophylactic antibiotic administration in patients undergoing endoscopic submucosal dissection and endoscopic submucosal excavation. The aim of this study was to prospectively evaluate the frequency of bacteremia and associated complications after endoscopic submucosal excavation.

## 2. Patients and Methods

One hundred and twenty-two consecutive patients with gastric muscular layer tumor enrolled in our study (60 women, 62 men; median age 45 years, range 22 to 74 years). All patients signed informed consent before the procedure. Exclusion criteria were the following: (1) bacterial infection and/or antibiotic treatment within 3 weeks; (2) need for antibiotic prophylaxis according to the American Heart Association Guidelines; (3) immune deficiency status; (4) age less than 18 years; (5) occurrence of contraindications for endoscopy procedures; (6) perforation of gastric wall during ESE procedure; and (7) lack of informed consent. This study was approved by the ethics committee of our hospital.

The indications for ESE were the following: (1) the tumor located in the gastric muscular layer; (2) the diameter of mass ranged from 5 mm to 30 mm; and (3) the mass showed hypoecho under ultrasonic endoscopy.

## 3. Microbiologic Examination

Immediately before and 15 minutes after ESE, 12 mL of blood was drawn by separate peripheral venipuncture by the same examiner. The specimens were equally distributed into an aerobic blood culture bottle and an anaerobic blood culture bottle (BAcT/ALERT 3D). Venipuncture was performed under a germ-free condition after the skin was sterilized with povidone-iodine and left at least 30 seconds for bactericidal effect. Blood cultures were incubated at 35°C for 5 days. If there was bacterial growth, the microorganisms were identified and tested for antibiotic sensitivity. All patients with a positive culture result had observed clinical signs of infection for at least 5 further days.

## 4. ESE Procedure and Treatment after ESE

Prior to an endoscopic submucosal excavation, all lesions were confirmed to be originated from muscularis propria by EUS (GF-UC240P-AL5, Olympus). Endoscopic submucosal excavation procedure was performed under intravenous anesthesia with propofol (GF-UC240P-AL5, Olympus) according to the following steps. The superficial mucosa of the lesion was marked with APC (40 W soft coagulation) to determine the location of the tumor. A salt solution containing 0.005 mg/mL epinephrine and 0.1% indigo carmine was injected into the submucosa. After sufficient lifting, a hook knife (KD 620LR, Olympus) was used to cut the superficial mucosa open along a straight line. The separation of the submucosal layer and the tumor was performed carefully with IT knife-2 (KD 611L, Olympus) under the endocut mode of electrosurgical accessories (ICC300, Erbe Co.). To avoid bleeding, small vessels were coagulated directly by knives; large vessels with high bleeding risk were coagulated with hemostatic forceps (Olympus). The body of the tumor was gradually exposed when the incision was wide enough. The muscularis propria tumor was snared or continued to be dissected by the IT knife after complete exposure of the root of the tumor. After resection of the tumor, the incision was closed by clips. The stomach tube was inserted into stomach for at least 24 h. The specimen was collected and sent to pathology department. The patients received transfusion and PPI after being transported to ward. The patients fasted for at least 24 h and then removed the stomach tube and took liquid or semifluid for 7 days. No symptoms and signs were observed in all patients for at least the following 7 days.

## 5. Results

All tumors that originated from muscularis propria were removed from 122 patients. The median size was 10 ± 2.7 mm (range from 5 mm to 30 mm). The median operation time was 29 ± 9.4 min (range from 8 min to 62 min). The locations of the tumors in fundus, body of stomach, and antrum were 24.6% (30/122), 65.6% (80/122), 9.8% (12/122), respectively. There were 83 stromal tumors, 37 leiomyomas, and 2 neurinomas. All patients had no symptoms and signs of peritonitis.

The blood cultures of 244 samples from 122 patients were performed before and 15 min after ESE. There were nine positive culture samples from 9 patients' blood samples 15 minutes after ESE. The results are listed in Table 1. Among nine positive blood culture samples, there were four kinds of* Staphylococcus* with coagulase negative from six samples. The six positive blood cultures were considered to be contaminant. There were two samples with gram positive bacilli and one sample with* Micrococcus luteus*. All patients remained asymptomatic without fever (defined as an oral temperature of 38°C) or chills during the observation period in gastroenterology department. There was no infectious complication (e.g., fever, phlegmon, or abscess formation) reported by any subjects or the referring physicians at one week after ESE.

## 6. Discussion

In daily living, transient bacterial seeding of the blood commonly may appear while brushing tooth and chewing food [20]. Bacteremia develops also commonly after some operation (e.g., nasogastric intubation, digital rectal examination, barium enema, and uncomplicated vaginal delivery) [20–25]. Antibiotic prophylaxis is clearly not indicated in these settings because of the limited duration (less than 15 minutes) of bacteremia and lack of clinical sequelae.

The longer time bacteremia can lead to bacterial endocarditis in some high-risk or moderate-risk patients. The AHA and ASGE recommended prophylactic administration of antibiotics for patients with high-risk cardiac conditions who are undergoing high-risk procedures [1–3].

Until recently, there were rare studies about bacteremia incidence after ESD [26–28]. Itaba et al. [26] reported that the bacteremia incidence was 4.4% (2/45) before gastric ESD, 4.3% (2/46) 10 min after gastric ESD, and 0% (0/46) 3 h after gastric ESD. Kato et al. [27] reported that the bacteremia incidence was 2% (2/100) after gastric ESD. So the bacteremia incidence may be low and transient after gastric ESD. In their studies, most gastric lesions originated from mucosa layer or submucosa layer; fewer lesions originated from muscular layer. But there was no study about the bacteremia incidence after ESE for gastric muscularis propria tumors. This study researched prospectively the bacteremia incidence after ESE for gastric muscularis propria tumors.

In the present study, bacteremia developed in three patients (2.5%, 3/122) 15 minutes after ESE. Six patients grew coagulase-negative* Staphylococcus* (*n* = 6), which deemed contaminants, not typically associated with ESE. All samples grew no bacteria before ESE. There was no evidence of immediate or delayed infection complications in any patients with positive blood cultures (actual or contaminant) after ESE. So bacteremia incidence was low after ESE and may be transient because of all patients without infection complications. Why coagulase-negative* Staphylococcus* was taken for contaminant bacteria in this paper? The coagulase-negative* Staphylococcus* is the most common flora in skin, and the most common contaminant bacteria in blood culture. There is a few coagulase-negative* Staphylococcus* in upper gastrointestinal tract of normal human. It is scarcely possible that endoscope was contaminated by coagulase-negative* Staphylococcus* in skin because the endoscope was sterilized by orthophthalaldehyde before ESE, and the operator abided by aseptic technique during procedure. It is also scarcely possible that the endoscope was contaminated by upper gastrointestinal tract flora because the blood culture was single coagulase-negative* Staphylococcus* in the 6 patients. Moreover, the six patients with coagulase-negative* Staphylococcus* blood culture had not any infection symptoms. Thereby we took coagulase-negative* Staphylococcus* for contaminant bacteria as other studies [26, 27].

The operation time and size of tumor may affect the bacteremia incidence after gastric ESE. In the present study, the diameter of tumor ranged from 5 mm to 30 mm. We select those sizes of tumors to treat for the following reasons. (1) The most muscularis propria tumor of stomach was stromal tumor. Malignant degree may increase if the diameter more than 30 mm, which may be not proper to remove by ESE. (2) The ESE for treating these tumors might have infrequent complication and less operation time. The median operation time was 29 min (from 8 min to 62 min). So it was safe and efficient that endoscopic excavation of these size tumors. In the patients with positive blood culture, the median operation time was 31 min (from 28 min to 35 min), and mean size was 10.7 mm. The median operation time and the mean size of patients with positive blood culture were similar to those in patients with negative blood culture. So the positive blood cultures were not related to tumor size (less than 30 mm) and operation time.

The perforation of stomach may affect the bacteremia incidence during ESE procedure. The abdominal cavity of those patients with perforation may be contaminated by gastrointestinal bacteria. These patients should administrate prophylactic antibiotics. So we excluded these patients with perforation in our study.

In summary, bacteremia develops after ESE of gastric muscularis propria tumors (less than 30 mm) at a low rate similar to that for diagnostic endoscopy examination. ESE of gastric muscularis propria tumors (less than 30 mm) should be considered a low-risk procedure for infection complications, which does not warrant the prophylactic administration of antibiotics for the prevention of endocarditis.

## Figures and Tables

**Table 1:** The positive blood culture results.

Number	Location of the tumor	Before ESE	Fifteen minutes after ESE	Operation time	Size (cm)
Number 1	Fundus	Negative	*Staphylococcus capitis *	28 min	0.6
Number 2	Body of stomach	Negative	*Staphylococcus capitis *	35 min	1.3
Number 3	Fundus	Negative	*Staphylococcus warmen *	34 min	1.2
Number 4	Fundus	Negative	*Staphylococcus warmen *	28 min	0.9
Number 5	Body of stomach	Negative	*Staphylococcus hominis *	29 min	2.5
Number 6	Body of stomach	Negative	*Staphylococcus cohnii *	31 min	1.0
Number 7	Fundus	Negative	Gram positive bacilli	32 min	0.5
Number 8	Body of stomach	Negative	Gram positive bacilli	29 min	0.8
Number 9	Body of stomach	Negative	*Micrococcus luteus *	31 min	0.8
